# High accuracy differentiating autoimmune pancreatitis from pancreatic ductal adenocarcinoma by immunoglobulin G glycosylation

**DOI:** 10.1186/s12014-018-9221-1

**Published:** 2019-01-03

**Authors:** Hsi-Chang Shih, Ming-Chu Chang, Chein-Hung Chen, I-Lin Tsai, San-Yuan Wang, Ya-Po Kuo, Chung-Hsuan Chen, Yu-Ting Chang

**Affiliations:** 1 Department of Chemistry, National Taiwan University, Taipei, Taiwan; 2 The Genomics Research Center, Academia Sinica, Taipei, Taiwan; 3 Department of Internal Medicine, National Taiwan University Hospital, College of Medicine, National Taiwan University, No. 7 Chung Shan South Road, Taipei, 100 Taiwan; 4 Department of Biochemistry and Molecular Cell Biology, School of Medicine, College of Medicine, Taipei Medical University, Taipei, Taiwan; 5 Graduate Institute of Medical Sciences, College of Medicine, Taipei Medical University, Taipei, Taiwan; 6 Master Program in Clinical Pharmacogenomics and Pharmacoproteomics, College of Pharmacy, Taipei Medical University, Taipei, Taiwan

**Keywords:** Autoimmune pancreatitis (AIP), Pancreatic cancer, Glycosylation, Immunoglobulin G (IgG)

## Abstract

**Background:**

Misdiagnosis of autoimmune pancreatitis (AIP) as pancreatic cancer (PDAC) or vice versa can cause dismal patents’ outcomes. Changes in IgG glycosylation are associated with cancers and autoimmune diseases. This study investigated the IgG glycosylation profiles as diagnostic and prognostic biomarkers in PDAC and AIP.

**Methods:**

Serum IgG-glycosylation profiles from 86 AIP patients, 115 PDAC patients, and 57 controls were analyzed using liquid chromatography–electrospray ionization mass spectrometry. Classification and regression tree (CART) analysis was applied to build a decision tree for discriminating PDAC from AIP. The result was validated in an independent cohort.

**Results:**

Compared with AIP patients and controls, PDAC patients had significantly higher agalactosylation, lower fucosylation, and sialylation of IgG1, a higher agalactosylation ratio of IgG1 and a higher agalactosylation ratio of IgG2. AIP patients had significantly higher fucosylation of IgG1 and a higher sialylation ratio of IgG subclasses 1, 2 and 4. Using the CART analysis of agalactosylation and sialylation ratios in the IgG to discriminate AIP from PDAC, the diagnostic accuracy of the glycan markers was 93.8% with 94.6% sensitivity and 92.9% specificity. There were no statistically significant difference of IgG-glycosylation profiles between diffuse type and focal type AIP.

**Conclusions:**

AIP and PDAC patients have distinct IgG-glycosylation profilings. IgG-glycosylation could different PDAC from AIP with high accuracy.

**Electronic supplementary material:**

The online version of this article (10.1186/s12014-018-9221-1) contains supplementary material, which is available to authorized users.

## Introduction

Differential diagnosis between autoimmune pancreatitis (AIP) and pancreatic ductal adenocarcinoma (PDAC) can be very difficult. Immunoglobulin G (IgG)4-related AIP is the most frequently recognized manifestation of IgG4-related disease, characterized clinically by obstructive jaundice, morphologically by diffuse or focal enlargement of the pancreas and irregular narrowing of the main pancreatic duct, serologically by elevated serum IgG and/or IgG4 levels, and therapeutically by a dramatic response to steroid treatment [1]. AIP typically presents as pseudo-tumor-like lesions in the pancreas, and many patients are misdiagnosed initially as having PDAC. Elevated serum IgG4 levels are characteristic of AIP. However, elevated serum IgG4 levels are also detected in PDAC [2]. Therefore, serum IgG4 concentrations lack adequate sensitivity and specificity for diagnostic purposes [2]. The mistaken diagnosis of AIP as PDAC can result in unnecessary surgery with risks of mortality and morbidity. The mistaken diagnosis of PDAC as AIP can result in delayed treatment and can impair the patient’s survival. To date, there is no simple serum biomarker, including IgG4 and CA19-9, that can be used as confirmative indicator of either disease.

Glycosylation is one of the most ubiquitous post-translational modifications. Human immunoglobulin G, sub-grouped into four subclasses (IgG1–IgG4), is the most abundant glycoprotein in serum. The heavy chain of IgG contains a branched glycan moiety attached to the asparagine 297 residue in the Fc region. IgG-Fc glycan is an essential functional structure for the binding of IgG with FcγR [3, 4]. Alterations in IgG glycan composition significantly affect their functions, including the activation of complement, the formation of immune complexes, and antibody dependent cellular cytotoxic activity (ADCC) [3, 5, 6]. Changes in IgG glycans are associated with aging, cancers, autoimmune diseases, and infections [7–10]. Previous studies have shown that high galactosylation of IgG1 in immune complexes exert anti-inflammatory properties [11]. The lack of core fucose and increased level of bisecting GlcNAc enhance ADCC [3, 4, 12, 13]. The presence of sialic acid with 2,6-linkages to the galactose on the Fc N-glycans leads to the functional shift from pro-inflammatory to anti-inflammatory [14]. The glycosylation of IgGs in AIP has not been studied and the roles of the IgG glycans in AIP and PDAC are not well understood. Liquid chromatography–electrospray ionization mass spectrometry (LC–ESI–MS/MS) with purification via hydrophilic interaction liquid chromatography (HILIC) has been a suitable method for IgG glycosylation profiling [15, 16]. The HILIC method provides high resolution and selectivity for many glycan isomers. The aims of this study are to investigate the IgG glycosylation profiles in patients with AIP and PDAC and to combine LC–MS and classification and regression tree (CART) analysis to develop decision rules for the differential diagnosis of PDAC from AIP.

## Methods

### Study populations

Between January 1996 and December 2012, peripheral blood was collected from a total of 86 AIP patients and 115 patients with cytologically and/or pathologically confirmed adenocarcinoma of the pancreas after obtaining their written informed consent at the National Taiwan University Hospital. All patients with AIP fulfilled the International Consensus Diagnostic Criteria (ICDC) [17] for type 1 AIP. The AIP patients had a follow-up period of at least 3 years (median 78.7 months; range 14.1–120.9 months) except one patient who died of septic shock in the 14th month after diagnosis. No patients with AIP presented malignancy. All the patients’ demographic data, including age, gender, serological studies, image studies, survival data, and clinical manifestations were collected (Additional file 1: Table S1). Peripheral blood was collected from 57 control subjects who underwent a health examination without evidence of AIP or malignancy during the study period. The study was approved by the Institutional Review Board of National Taiwan University Hospital.

### IgG purification and HILIC solid phase extraction

In the discovery phase, a total of 86 AIP, 115 PDAC patients, and 57 control individuals were analysed. The IgG purification and tryptic digestion from sera were modified from the method published by Selman et al. [18]. Briefly, IgG subclasses were captured using recombinant Protein A beads (TOSOH Corporation, Tokyo, Japan) from 2 μL of serum. Purified IgGs were digested with 20 μL of 0.02 μg/μL trypsin (Promega, Madison, WI, USA) at 37 °C overnight. Eight microliters of Sepharose Cl-4B beads (GE Healthcare, Uppsala, Sweden) was activated and sequentially conditioned with water and 83% acetonitrile (ACN). The IgG digests and beads were mixed and incubated in 83% ACN at room temperature for 30 min. The bead-captured IgG glycopeptides were washed twice in 83% ACN containing 0.1% trifluoroacetic acid (TFA) and twice sequentially in 83% ACN. The enriched glycopeptides were eluted with double-distilled water for MS analysis.

### LC–ESI–MS/MS analysis

An LTQ-Orbitrap XL ETD MS with a nanoelectrospray ion source (New Objective, Inc., Woburn, MA, USA) and an UltiMate 3000 Nano LC System (Thermo Fisher Scientific, San Jose, CA, USA) were applied for glycosylation profiling of the pooled sera (Additional file 1: Supplementary methods). A Velos Pro mass spectrometer (Thermo Fisher Scientific) with a standard ESI ion source and Accela LC system (Thermo Fisher Scientific) was applied for glycosylation profiling for individual sera (Additional file 1: Supplementary methods). The triple-protonated signals (m/z) of the IgG glycopeptides with glycoforms G0F, G1F, and G2FS of IgG1, IgG2 and IgG4 (Additional file 1: Table S2) were targeted fragmentation using CID. Mixed serum from the control group was used as a quality control (QC) analyzed at the beginning of an analysis. To determine the precision/repeatability of the analytical method, the experiments were performed in triplicates on three different days.

### Data processing

The measured masses of peptides and glycopeptides were compared with databases containing the prediction of tryptic peptides (Protein Digest Simulator Basic) and N-linked glycans (Consortium for Functional Glycomics) by in-house software [19]. Glycoforms abundances in each sample were processed and evaluated by Xcalibur software ver. 2.2 SP1.48 (Thermo Fisher Scientific) and MS/MS Automated Selected Ion Chromatogram Generator (MASIC) software [20]. Quantification of glycosylation of IgG was accomplished by generating extracted ion chromatograms (XIC) for specific IgG glycopeptides, and the resulting peak areas were used for relative quantification of glycosylation species (Additional file 1: Figure S1). The theoretical 16 most abundant glycoforms of IgG1, IgG2, and IgG4 are listed in Table S2. The relative abundances of galactosylation, agalactosylation, sialylation, bisecting GlcNAc, and fucosylation of IgG1 were calculated according to the following formulas, which were normalized to the total abundances of IgG1 glycoforms. Galactosylation: (G1 + G1F + G1FN + G1N + G1S + G1FS) * 0.5 + G2 + G2F + G2FN + G2N + G2S + G2FS. Agalactosylation: G0F + G0FN + G0N + G0. The bisecting GlcNAc: G0N + G1N + G2N + G0FN + G1FN + G2FN. Sialylation: G1S + G1FS + G2S + G2FS. Fucosylation: G0F + G0FN + G1F + G1FN + G1FS + G2F + G2FN + G2FS.

The partial least square-discriminate analysis (PLS-DA) [21] was used to estimate how well the IgG glyans could distinguish the PDAC from AIP and control. The variables (IgG glycoforms and IgG glycosylation features) were selected based on the variable importance in the projection (VIP) scores obtained from the PLS-DA model [22]. After PLS-DA, the IgG glycoforms (G0F, G1F, and G2FS of IgG1, IgG2 and IgG4) were selected as discriminating variables among the AIP, PDAC, and controls. Herein, the G0F/G1F ratio and the G2FS/G1F ratio were used as the agalactosylation and sialylation ratios. The candidate IgG glycans were used to develop decision rules using the CART approaches [23].

### Validation

An independent validation cohort composed of 28 AIP and 37 PDAC patients were used to validate glycan profiling as differential markers for AIP and PDAC. The diagnostic accuracy, sensitivity, and specificity of the glycan markers were calculated based on CART model.

### Statistical analysis

The differences of IgG glycosylation among the AIP, PDAC, and controls were assessed by one-way and two-way analyses of variance (ANOVAs) with Bonferroni tests for multiple comparisons using GraphPad Prism version 7 for windows (GraphPad Software, La Jolla, CA, USA). All statistical tests were two-tailed. The Kaplan–Meier test was used for survival analysis. The log-rank test was applied to compare survival between subgroups. The variables of glycoforms, age, gender, tumor stage, and chemotherapy were subjected to univariate and multivariate Cox proportional hazards regression analysis for survival. P < 0.05 was considered significant. The analyses were performed using SPSS software package version 17 (SPSS, Chicago, IL, USA). PLS-DA and CART were performed using R software (http://www.r-project.org/) with packages of “mixOmics” and “rpart.plot” respectively.

## Results

### IgG glycosylation profiling

A total of sixteen glycoforms of the IgG subclasses had been observed in the pooled and individual sera. The quantity of the eleven most abundant glycoforms accounted for more than 96% of the total glycans (Additional file 1: Figure S2). In PLS-DA model, the G0F of IgG1 and IgG4, G1F of IgG1 and IgG4, and G0F of IgG2 with the highest VIP scores (relative abundance > 5%) are the most contributory variables to differentiate PDAC from AIP (Additional file 1: Table S3). The intraday repeatability of the analytical method was less than 11% of relative standard deviation (RSD), and the interday RSD was below 15% (Additional file 1: Table S4).

### Increased fucosylation and sialylation in AIP and agalactosylation in PDAC

The summary of glycosylation changes compared with controls in AIP and PDAC are listed in the Table 1. Agalactosylation of IgG1 and agalactosylation ratio of IgG1 and IgG2 were significantly higher in PDAC patients than those in AIP patients and controls. The fucosylation of IgG1, sialylation ratio of IgG1, IgG2, and IgG4 were significantly increased in AIP patients, while the bisecting GlcNAc of IgG1 was significantly decreased than those in PDAC patients and controls. Agalactosylation ratio of IgG4 in both AIP and PDAC patients were higher than controls (Additional file 1: Figure S3-I and II). There was no diffenerce of the IgG glycoforms between the focal and diffuse subtypes of AIP (Additional file 1: Figure S4-I and II).

**Table 1:** Summary of glycosylation changes in autoimmune pancreatitis (AIP) and pancreatic ductal adenocarcinoma (PDAC)

Glycoforms	AIP versus control	*P* value	AIP versus PDAC	P-value	PDAC versus control	P-value
Fucosylation of IgG1	↑	P < 0.001	↑	P < 0.001	↓	P < 0.001
Bisecting GlcNAc of IgG1	↓	P < 0.001	↓	P < 0.001	NS	–
Agalactosylation of IgG1	NS	–	↓	P < 0.001	↑	P < 0.001
Sialylation of IgG1	NS	–	↑	P < 0.001	↓	P < 0.05
Galactosylation of IgG1	NS	–	↑	P < 0.001	NS	–
Sialylation ratio of IgG1	↑	P < 0.05	↑	P < 0.001	NS	–
Sialylation ratio of IgG2	↑	P < 0.001	↑	P < 0.001	NS	–
Sialylation ratio of IgG4	↑	P < 0.001	↑	P < 0.001	NS	–
Agalactosylation ratio of IgG1	NS	–	↓	P < 0.05	↑	P < 0.001
Agalactosylation ratio of IgG2	NS	–	↓	P < 0.001	↑	P < 0.01
Agalactosylation ratio of IgG4	↑	P < 0.001	NS	–	↑	P < 0.001

↑: increased; ↓: decreased; –: P ≧ 0.05; NS: not significant

### Agalactosylation ratios and sialylation ratios of the IgG as markers to differentiate PDAC from AIP

All of 19 variables in discovery cohort, including glycofeatures of IgG1, agalactosylation and sialylation ratios, and the sums of the agalactosylation and sialylation ratios in the IgG subclasses were subjected to the PLS-DA. The variables with the highest VIP score and maximum area under ROC curve (AUC) are considered as the potentially differential markers (Table 2). The sum of the sialylation ratios of IgG2 and IgG4 at a cutoff value of 1.355 had a sensitivity of 77% and a specificity of 80% to distinguish PDA from AIP patients (VIP = 1.57; AUC = 0.86). The sum of the sialylation ratios of IgG2 and IgG4 at a cutoff value of 1.356 yielded a sensitivity of 80% and a specificity of 84% to differentiate AIP from controls (VIP = 1.56; AUC = 0.87). The sum of the agalactosylation ratios of IgG1 and IgG4 at a cutoff value of 1.983 led to a sensitivity of 65% and a specificity of 86% to differentiate PDAC patients from controls (VIP = 1.71; AUC = 0.82) (Table 2 and Additional file 1: Figure S5). There were no statistically significant differences of the sum of sialylation ratios between the AIP patients with different serum IgG4 concentrations (Additional file 1: Figure S6A-B).

**Table 2:** The P-value, area under ROC curve (AUC) and importance in the projection (VIP) of IgG-Fc N-glycans in IgG subclasses for discriminating among AIP patients, PDAC patients, and control

Glycoform	PDAC versus control			AIP versus control			AIP versus PDAC		
	P	AUC	VIP	P	AUC	VIP	P	AUC	VIP
Agalactosylation ratio of IgG1	< 0.0001****	0.7542	1.29	0.0776	0.5873	0.66	0.0009***	0.6371	0.48
Agalactosylation ratio of IgG2	0.002**	0.6449	0.95	0.4859	0.5345	0.05	0.0004***	0.6454	0.65
Agalactosylation ratio of IgG4	< 0.0001****	0.7913	1.61	< 0.0001****	0.7729	1.22	0.6415	0.5192	0.05
Agalactosylation ratio of IgG1 and agalactosylation ratio of IgG2	< 0.0001****	0.732	1.28	0.5582	0.529	0.43	0.0001***	0.6581	0.61
Agalactosylation ratio of IgG1 and agalactosylation ratio of IgG4	< 0.0001****	*0.8186*	*1.71*	< 0.0001****	0.7258	1.12	0.0618	0.577	0.26
Agalactosylation ratio of IgG2 and agalactosylation ratio of IgG4	< 0.0001****	0.7805	1.58	< 0.0001****	0.6938	1.00	0.0508	0.5806	0.30
Agalactosylation ratio of IgG1, Agalactosylation ratio of IgG2, and Agalactosylation ratio of IgG4	< 0.0001****	0.8041	1.64	0.0009***	0.6636	0.95	0.0094**	0.6072	0.40
Sialylation ratio of IgG1	0.0212*	0.6081	0.48	0.0128*	0.6232	0.65	< 0.0001****	0.6963	0.92
Sialylation ratio of IgG2	0.8262	0.5103	0.00	< 0.0001****	0.777	1.22	< 0.0001****	0.7625	1.25
Sialylation ratio of IgG4	0.2838	0.5503	0.03	< 0.0001****	0.8641	1.49	< 0.0001****	0.8507	1.46
Sialylation ratio of IgG1 and sialylation ratio of IgG2	0.2666	0.5521	0.32	< 0.0001****	0.7217	1.03	< 0.0001****	0.7483	1.20
Sialylation ratio of IgG1 and sialylation ratio of IgG4	0.0543	0.5902	0.28	< 0.0001****	0.8054	1.32	< 0.0001****	0.8265	1.39
Sialylation ratio of IgG2 and sialylation ratio of IgG4	0.7685	0.5138	0.02	< 0.0001****	*0.8676*	*1.56*	< 0.0001****	*0.8559*	*1.57*
Sialylation ratio of IgG1, sialylation ratio of IgG2, and sialylation ratio of IgG4	0.3332	0.5454	0.23	< 0.0001****	0.8252	1.39	< 0.0001****	0.8317	1.47
Fucosylation of IgG1	0.0012**	0.6522	1.01	< 0.0001****	0.714	1.00	< 0.0001****	0.8079	1.40
Bisecting GlcNAc of IgG1	0.9494	0.503	0.15	< 0.0001****	0.696	0.99	< 0.0001****	0.7068	0.91
Agalactosylation of IgG1	< 0.0001****	0.7458	1.30	0.3493	0.5463	0.10	< 0.0001****	0.7395	0.95
Sialylation of IgG1	0.1926	0.5611	0.48	0.0241*	0.6116	0.58	< 0.0001****	0.6832	0.87
Galactosylation of IgG1	0.0016**	0.6481	0.80	0.1124	0.5785	0.39	< 0.0001****	0.6931	0.83

Controls (n = 57), PDAC (n = 115), and AIP (n = 86)

P-values: *P < 0.05; **P < 0.01; ***P < 0.001; ****P < 0.0001

Italic values indicate the p-value <  0.0001 and the variables with the highest VIP score and maximum area under ROC curve (AUC) to differentiate AIP from PDAC patients, AIP or PDAC patients from controls

### CART analysis

**Fig. 1:**
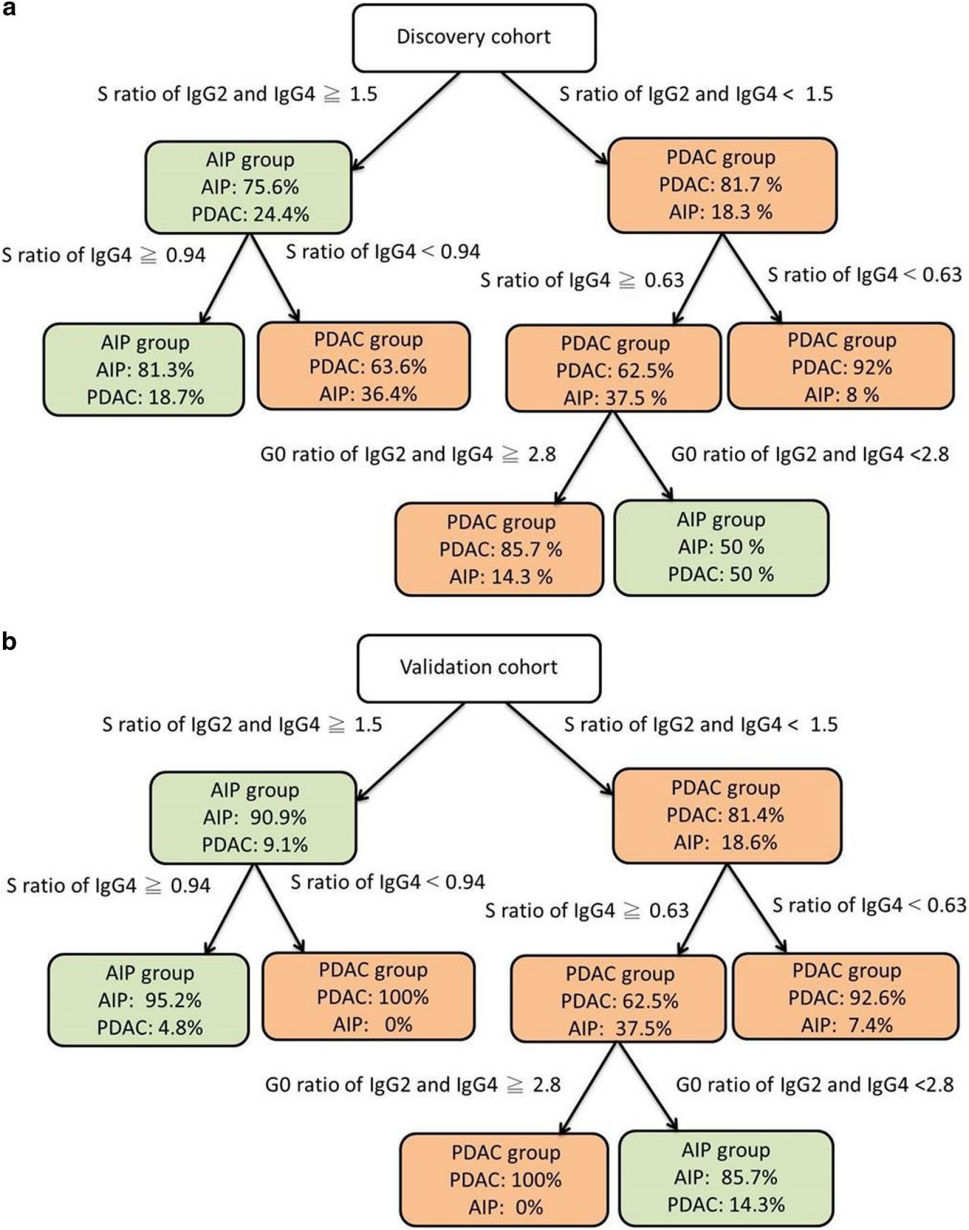
Classification and regression tree (CART) model in differentiating autoimmune pancreatitis (AIP) from pancreatic ductal adenocarcinoma (PDAC) patients. The AIP and PDAC groups are shown in the green- and red-colored boxes respectively. The probability are given inside each node. **a** CART decision tree of the discovery cohort. **b** CART decision tree of the validation cohort

The CART model generated from the discovery cohort (Fig. 1a) was verified by an independent validation cohort (Fig. 1b). The sialylation ratios of IgG2 and IgG4 at cutoff of 1.5 was the first node for further evaluation. Using the sialylation ratios of IgG4 cutoff less than 0.63, PDAC was detected in 92.6% of cases (25 of 27). In subjects with sialylation ratios of IgG4 greater than 0.63, PDAC was detected in 100% (9 of 9) when the sum of the agalactosylation ratios of IgG2 and IgG4 greater than 2.8. AIP was detected in 85.7% (6 of 7) when the sum of the agalactosylation ratios of IgG2 and IgG4 less than 2.8. AIP was detected in 95.2% (20 of 21) when the sialylation ratios of IgG4 greater than 0.94, and no AIP was detected when the sialylation ratios of IgG4 less than 0.94. The overall CART model was correctly identified 26 out of the 28 AIP patients and 35 out of the 37 PDAC patients with 94.6% sensitivity, 92.9% specificity and 93.8% accuracy (Fig. 1).

### IgG glycoforms in AIP

There were 74 (86.0%) AIP patients with extrapancreatic manifestations, including 31 (36.1%) with hepatobiliary involvement. Compared to the AIP patients without hepatobiliary involvement, the AIP patients with hepatobiliary involvement had higher agalactosylation and sialylation, and lower galactosylation of IgG1 (Table 3).

**Table 3:** Glycoforms in the autoimmune pancreatitis patients with and without hepatobiliary involvement

Glycoform	Without hepatobiliary involvement	With hepatobiliary involvement	P
Galactosylation of IgG1 (%)	62.52 ± 5.73	59.33 ± 1.39	0.032*
Agalactosylation of IgG1 (%)	15.49 ± 4.63	18.56 ± 6.23	0.015*
Bisected glycoforms of IgG1 (%)	26.48 ± 5.87	29.42 ± 7.61	0.061
Sialylation of IgG1 (%)	20.49 ± 4.86	21.45 ± 8.71	0.481
Fucosylated of IgG1 (%)	85.86 ± 3.51	85.95 ± 3.69	0.920
Agalactosylation ratio			
Agalactosylation ratio of IgG1	0.83 ± 0.43	1.06 ± 0.54	0.042*
Agalactosylation ratio of IgG2	0.79 ± 0.33	0.98 ± 0.36	0.021*
Agalactosylation ratio of IgG4	1.41 ± 0.84	1.35 ± 0.62	0.721
Sialylation ratio			
Sialylation ratio of IgG1	0.78 ± 0.36	1.00 ± 0.57	0.038*
Sialylation ratio of IgG2	0.72 ± 0.42	0.78 ± 0.41	0.540
Sialylation ratio of IgG4	1.48 ± 0.76	1.83 ± 1.21	0.017*
Summation			
Agalactosylation ratio of IgG1 and agalactosylation ratio of IgG4	0.24 ± 0.15	2.54 ± 0.24	0.289
Sialylation ratio of IgG2 and sialylation ratio of IgG4	2.07 ± 0.11	2.21 ± 0.12	0.038*
Sialylation ratio of IgG1, sialylation ratio of IgG2, and sialylation ratio of IgG4	2.8 ± 0.14	3.05 ± 0.15	0.028*

Galactosylation (%): (G1 + G1F + G1FN + G1N + G1S + G1FS1) * 0.5 + G2 + G2F + G2FN + G2N + G2S1 + G2/total IgG1; agalactosylation (%): G0F + G0FN + G0N + G0/total IgG1; bisected glycoforms (%): G0N + G1N + G2N + G0FN + G1FN + G2FN/total IgG1; sialylation of IgG1 (%): G1FS1 + G2S1 + G2FS1 + G1S/total IgG1; fucosylation (%): G0F + G0FN + G1F + G1FN + G1FS1 + G2F + G2FN + G2FS1/total IgG1

*P < 0.05; **P < 0.01; ***P < 0.001

### IgG glycoforms in PDAC

Compared with early stage of PDAC patients, the advanced stage PDAC patients had higher agalactosylation and sialylation, and lower galactosylation of IgG1 (Table 4). The top 25% of PDAC patients survived 34.3 months in stage I, 22.6 in stage II, 11.9 in stage III, and 5.5 in stage IV. Compared with the ordinary survival of PDAC patients, the PDAC patients with the top 25% of survival times in each stage had significantly lower fucosylation of IgG1 (Table 4). In univariate and multivariate analyses, stage, chemotherapy, and fucosylation of IgG1 are independent predictors of longer survival in PDAC patients (Table 5).

**Table 4:** Glycoforms in the early and advanced stages of pancreatic ductal adenocarcinoma

Glycoforms	Cancer stage			Patients’ survival		
	Early	Advanced	P	Top 25% percentile of survival	Ordinary	P
Galactosylation of IgG1 (%)	61.66 ± 5.26	58.02 ± 4.07	0.007*	59.67 ± 4.16	57.93 ± 4.29	0.060
Agalactosylation of IgG1 (%)	17.38 ± 4.54	20.24 ± 4.42	0.044*	19.24 ± 4.31	20.21 ± 4.55	0.317
Bisected glycoforms of IgG1 (%)	29.58 ± 6.42	31.81 ± 5.71	0.226	30.21 ± 5.35	32.06 ± 5.89	0.140
Sialylation of IgG1 (%)	16.97 ± 5.47	17.54 ± 4.13	0.673	17.61 ± 4.22	17.44 ± 4.29	0.853
Fucosylated of IgG1 (%)	79.40 ± 6.32	81.17 ± 4.17	0.208	79.50 ± 3.97	81.50 ± 4.46	0.034*
Agalactosylation ratio						
Agalactosylation ratio of IgG1	0.93 ± 0.30	1.10 ± 0.62	0.371	0.10 ± 0.38	1.12 ± 0.65	0.351
Agalactosylation ratio of IgG2	1.01 ± 0.35	1.05 ± 0.48	0.789	1.01 ± 0.40	0.06 ± 0.49	0.651
Agalactosylation ratio of IgG4	1.08 ± 0.49	1.46 ± 0.80	0.090	1.34 ± 0.53	1.49 ± 0.85	0.370
Sialylation ratio						
Sialylation ratio of IgG1	0.55 ± 0.17	0.58 ± 0.33	0.764	0.49 ± 0.24	0.60 ± 0.33	0.080
Sialylation ratio of IgG2	0.42 ± 0.22	0.41 ± 0.20	0.865	0.35 ± 0.18	0.43 ± 0.20	0.053
Sialylation ratio of IgG4	0.81 ± 0.39	0.66 ± 0.42	0.277	0.71 ± 0.48	0.66 ± 0.39	0.641
Summation						
Agalactosylation ratio of IgG1 and agalactosylation ratio of IgG4	2.07 ± 0.25	2.50 ± 0.12	0.026*	2.51 ± 0.18	2.44 ± 0.14	0.782
Sialylation ratio of IgG2 and sialylation ratio of IgG4	1.57 ± 0.26	1.97 ± 0.11	0.025*	1.76 ± 0.18	1.99 ± 0.12	0.322
Sialylation ratio of IgG1, sialylation ratio of IgG2, and sialylation ratio of IgG4	2.28 ± 0.33	2.75 ± 0.14	0.030*	2.49 ± 0.23	2.77 ± 0.16	0.359

Early stage: stage I and II

Advanced stage: stage III and IV

Galactosylation of IgG1 (%): (G1 + G1F + G1FN + G1N + G1S + G1FS1) * 0.5 + G2 + G2F + G2FN + G2N + G2S1 + G2/total IgG1; agalactosylation of IgG1 (%): G0F + G0FN + G0N + G0/total IgG1

Bisected glycoforms of IgG1 (%): G0N + G1N + G2N + G0FN + G1FN + G2FN/total IgG1

Sialylation of IgG1 (%): G1FS1 + G2S1 + G2FS1 + G1S/total IgG1

Fucosylation of IgG1 (%): G0F + G0FN + G1F + G1FN + G1FS1 + G2F + G2FN + G2FS1/total IgG1

*P < 0.05; **P < 0.01; ***P < 0.001

**Table 5:** Univariate and multivariate analyses used to predict longer survival of patients with pancreatic ductal adenocarcinoma

Variate	Univariate analysis			Multivariate analysis		
	P value	OR	95% CI	P value	OR	95% CI
Age	0.119	0.97	0.94–1.01	0.665	0.99	0.95–.1.03
Gender	0.807	1.12	0.44–2.85	0.939	1.05	0.29–3.83
Diabetes	0.522	0.74	0.29–1.87	0.631	0.75	0.23–2.47
Smoking	0.668	1.24	0.47–3.27	0.453	1.66	0.44–6.23
Stage*^,#^	0.005	2.69	1.33–6.21	0.007	2.75	1.32–5.71
Chemotherapy*^,#^	0.003	5.95	1.84–19.29	0.003	8.86	2.10–37.54
Fucosylation*^,#^	0.015	0.86	0.76–0.97	0.033	0.79	0.64–0.98

Stage: early stage (stage I and II) versus advanced stage (stage III and IV)

*P < 0.05 in univariate analysis; ^#^P < 0.05 in multivariate analysis

## Discussion

Glycobiology has become increasingly important in cancer and autoimmune diseases, as it provides potential targets for diagnostic and therapeutic applications [24]. To our knowledge, this is the first report on the distinctive IgG-Fc glycosylation profiles among AIP and PDAC patients. Clinically, PDAC is a major differential diagnosis of AIP. The mistaken diagnosis of AIP as PDAC, or vice versa, can result in unnecessary surgery or delayed treatment and can impair the patient’s survival. To date, there is no simple serum biomarker, including serum IgG4 and/or CA19-9, that can be used a confirmative indicator of either disease. This study demonstrates that PDAC patients have higher agalactosylation and lower fucosylation and sialylation of IgG-Fc glycosylation. In contrast, AIP patients have significantly higher fucosylation of IgG-Fc glycosylation. A combination of a LC–MS IgG glycosylation profiling and CART analysis provided 94.6% sensitivity, 92.9% specificity, and 93.8% accuracy in distinguish PDAC from AIP patients, which is much better than the diagnostic accuracy (85.6%) with combination of serum IgG4 and CA19-9 levels [25]. The present study demonstrates for the first time that the quantitative analysis of IgG glycosylation can aid in the differential diagnosis of PDAC and AIP with high accuracy.

Decreased galactosylated IgG glycoforms have been reported in tumor progression and metastasis [26, 27]. The significant increase in the agalactosylation of IgG1 and the stage-dependent changes of IgG-Fc glycosylation observed in PDAC patients in this study were in line with previous reports of other cancers [10, 26–28]. The decrease in IgG-Fc sialylation in patients with PDAC was also consistent with earlier reports of patients with gastric [26] and ovarian cancer [28]. Patients in the advanced stages of PDAC exhibited significantly higher agalactosylation and lower galactosylation compared with patients in the early stages of PDAC, indicating that IgG glycosylation is related to cancer progression. Although Kodar et al. reported that agalactosylated IgG was associated with a lower survival rate in patients with gastric cancer [26], no relation between IgG galactosylation and the overall survival of PDAC patients was found in this study. Contrary to the absence of an association between IgG fucosylation and the survival of patients with gastric cancer [26], there was a significant decrease in IgG fucosylation of PDAC patients with the highest 25% of survival times. Increased core fucosylation has been reported during the process of hepatocarcinogenesis and cancer cell growth [19, 29]. In addition, the multivariable analysis revealed that chemotherapy and fucosylation are independent predictors of better survival in PDAC patients. The roles of different IgG glycoforms in the pathogenesis of PDAC require further studies.

IgG glycan composition involves the pathophysiology of autoimmunity [7, 30]. Maverakis et al. proposed “The Altered Glycan Theory of Autoimmunity”, which states that each autoimmune disease has a unique glycan signature, including the site-specific glycosylation patterns of the IgG [30]. In our study, AIP patients had significantly higher sialylation ratios of IgG1, IgG2, and IgG4 and increased fucosylation of IgG1 compared with the PDAC patients and controls. Sialylation has been proposed to have the greatest effect on the structure of the Fc domain of IgG, which closes the binding site for FcγRs and opens a cryptic binding site for dendritic cell-specific intercellular adhesion molecule-3-grabbing nonintegrin leading to anti-inflammatory activity [31, 32]. In contrast to other autoimmune diseases with decreased sialylated IgGs, AIP patients have a disease-specific glycan signature with higher sialylation ratios of IgG. The distinct changes in IgG-Fc glycans in sialylation in AIP patients may indicate that anti-inflammatory responses are activated. Increased agalactosylated IgG-Fc glycans in RA patients increases the affinity of pathogenic rheumatoid factors [33]. The IgG galactosylation ratio was correlated with RA severity [34]. We observed that both AIP and PDAC patients have a significantly higher agalactosylation ratio of IgG4 compared with the controls. A recent study for the whole-serum N-glycan profiles of AIP patients also demonstrated that the agalactosyl glycans were elevated in the AIP patients [35]. Elevated serum IgG4 in AIP patients has been proposed to dampen inappropriate inflammatory reactions because IgG4 can help to clear immune complexes or terminate the inflammatory process by preventing the formation of large immune complexes by blocking the Fc-mediated effector functions of IgG1 [36, 37]. The IgG4 antibodies in AIP patients may have both pathogenic and protective roles [38]. The roles of IgG4 glycosylation in the pathogenesis of AIP are still not well understood. Further studies are needed to elucidate the role of IgG4 glycosylation in the pathogenesis of AIP.

There are some limitations in this study. First, all AIP patients were type I AIP. Whether type II AIP patients harbor similar IgG-Fc glycosylation profiles requires further study. Second, the altered glycan profiles in the AIP and PDAC patients may be attributed to an under- or over-expression of sialidase, galactosyltransferase, or glycosyltransferase which are known to impact glycan structure [39]. We did not evaluate the enzymatic activities in this study. Third, the timing of measuring the IgG glycans after but not prior to the onset of disease indicates that what we observed the distinct IgG glycan profiling may not involve the initiation of disease but rather be related to the alteration of immunological response against cancer or autoimmune dysregulation. Finally, we were unable to conduct stratified analyses according to subtypes due to the insufficient sample size. However, there were no statistically significant differences of IgG Fc-glycolyation profiles between focal and diffuse type AIP. The combination of a LC–MS glycosylation profiling and CART analysis in this study provides a rapid and robust analysis of serum IgG Fc-glycosylation as promising differential diagnostic biomarkers in pancreatic disease for clinical application.

## Conclusions

In summary, distinct IgG Fc-glycosylation patterns were found among PDAC patients, AIP patients and controls. A quantitative analysis of IgG glycosylation profiling can aid in the differential diagnosis of PDAC from AIP with high accuracy. The rapid, robust, and accurate glycan analyses might aid in the better diagnosis and management of patients with PDAC or AIP.

## Additional file

**Additional file 1.** Supplementary Methods, Tables(S1–S4) and Figures (S1–S6).
