# Supplementary material for: High accuracy differentiating autoimmune pancreatitis from pancreatic ductal adenocarcinoma by immunoglobulin G glycosylation
Source: Clin Proteomics. 2019 Jan 3;16:1. doi: 10.1186/s12014-018-9221-1 (PMC6317216; doi:10.1186/s12014-018-9221-1)
Supplement: Supplementary file 1 — Additional file 1. Supplementary Methods, Tables(S1–S4) and Figures (S1–S6). [file 12014_2018_9221_MOESM1_ESM.docx]

**Additional file 1: Supplementary Methods. The experimental parameters of the mass spectrometer.
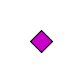

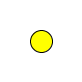

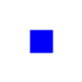

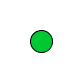
Table S1. Demographic data of the autoimmune pancreatitis (AIP) and pancreatic ductal adenocarcinoma (PDAC) patients and control subjects.** **Table S2. Theoretical masses of the tryptic glycopeptides of the human IgG subclasses 1, 2, and 4. Table S3.** **The variable importance in projection (VIP) score from PLS-DA to distinguish AIP, PDAC, and control. Table S4. Reproducibility of the analytical method for glycoform identification. Figure S1. Glycosylation profiling of IgG and relative quantification of G0F, G1F, and G2FS of IgG1, gG2, and IgG4. Figure S2. Glycosylation of IgG1, IgG2 and IgG4 in pooled sera from controls, AIP patients and PDAC patients. Figure S3-I. IgG1 Fc N-glycan (A) fucosylation, (B) bisecting GlcNAc, (C) agalactosylation, (D) sialylation, and (E) galactosylation in controls (n=57) and patients with PDAC (n=115) or AIP (n=86). Figure S3-II. The agalactosylation ratio and sialylation ratio of the three IgG subclasses (IgG1, IgG2 and IgG4) within the three groups of individual sera (controls, PDAC patients and AIP patients). Figure S4-I. IgG1 Fc N-glycan (A) fucosylation, (B) bisecting GlcNAc, (C) agalactosylation, (D) sialylation, and (E) galactosylation in diffuse type AIP patients (n=45) and focal type AIP patients (n=41). Figure S4-II. The agalactosylation ratio and sialylation ratio of the three IgG subclasses (IgG1, IgG2 and IgG4) in the diffuse type AIP patients (n=45) and focal type AIP patients (n=41). Figure S5. ROC curve for discrimination among the control, PDAC and AIP groups. Figure S6. The sialylation ratios in AIP patients with different serum IgG4 concentrations.**

**Supplementary Methods:**

The experimental parameters of the Linear Trap Quadropole -Orbitrap XL electron transfer dissociation (LTQ-Orbitrap XL ETD) mass spectrometer

Five microliters of enriched IgG glycopeptides were injected at a flow rate of 10 μL/min into a precolumn (150 μm I.D. × 30 mm, 5 μm, 200 Å). LC separation was performed on a self-packed C18 nanocolumn (75 μm I.D. × 200 mm, 3 μm, 200 Å), with 0.1% formic acid in water as mobile phase A and 0.1% formic acid in 80% acetonitrile as mobile phase B with a split flow rate of 300 nL/min. The full-scan mass range was set from *m*/*z* 320–2000 with a resolution of 30,000 with *m*/*z* at 400. The top three intense ions were sequentially isolated for collision-induced dissociation (CID) (resolution 7500). The capillary temperature was maintained at 200°C, and the electrospray voltage was set to 1.8 kV. The reproducibility of the experiments was determined from three independent experiments.

The experimental parameters of Velos Pro mass spectrometer

A Velos Pro mass spectrometer (Thermo Fisher Scientific, San Jose, CA, USA) with a standard ESI ion source and Accela LC system (Thermo Fisher Scientific, San Jose, CA, USA) was applied for glycosylation profiling for all individual sera. 5 µL of enriched IgG glycopeptides was injected at a flow rate of 100 μL/min. Chromatographic separation was performed using a reversed phase XBridge C18 column (1.0 mm I.D. × 150 mm, 3.5 μm, 130 Å, Waters Corporation, Milford, MA, USA). Buffer A (0.1% FA in water) and buffer B (0.1% FA in 80% ACN) were used as mobile phases for gradient separation. The 15 min gradient was 98% A, 2% B at 0-2 min; 90% A, 10% B at 3-9 min; 80% A, 20% B at 10 min; 2% A, 98% B at 11-12 min; 98% A, 2% B at 13-15 min. The top three intense ions were sequentially isolated for high-energy collision-induced dissociation (CID).

**
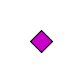

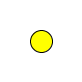

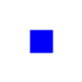

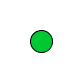
Supplementary Tables:**

**Table S1. Demographic data of the autoimmune pancreatitis (AIP) and pancreatic ductal adenocarcinoma (PDAC) patients and control subjects.**

| **Validation group** | AIP  (n=28) | PDAC  (n=37) | Stage I  (n=4) | Stage II  (n=7) | Stage III  (n=4) | Stage IV  (n=22) | Early  (n=11) | Advanced  (n=26) |
| --- | --- | --- | --- | --- | --- | --- | --- | --- |
| Male / Female | 24/4 | 25/12 | 2/2 | 5/2 | 2/2 | 16/6 | 7/4 | 18/8 |
| Age (Mean±SD) | 58.1±13.3 | 61.9±11.5 | 56.9±10.9 | 61.4±10.5 | 62.7±15.1 | 62.9±11.7 | 59.6±10.3 | 62.9±11.9 |

| **Study Population** | AIP  (n=86) | PDAC  (n=115) | Stage I  (n=2) | Stage II  (n=11) | Stage III  (n=28) | Stage IV  (n=74) | Early  (n=13) | Advanced  (n=102) | Control  (n=57) |
| --- | --- | --- | --- | --- | --- | --- | --- | --- | --- |
| Male / Female | 38/48 | 56/59 | 0/2 | 3/8 | 17/11 | 36/38 | 3/10 | 53/49 | 49/8 |
| Age (Mean±SD) | 50.8±15.9 | 64.6±13.4 | 53.4±7.1 | 63.7±13.1 | 63.3±11.1 | 65.6±14.3 | 62.1±12.8 | 64.9±13.5 | 68.0±4.5 |
| Survival (months, mean±SEM) |  | 8.4±0.9 | 26.9±7.8 | 19.0±4.6 | 12.9±1.4 | 4.5±0.6 | 20.2±4.0 | 6.8±0.7 |  |

AIP: autoimmune pancreatitis

PDAC: pancreatic ductal adenocarcinoma

Early stage: stage I and II

Advanced stage: stage III and IV

**
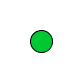

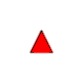

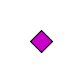

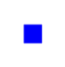

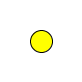
Table S2. Theoretical masses of the tryptic glycopeptides of the human IgG subclasses 1, 2, and 4.** The glycan species are given in terms of the number of galactose (G0, G1, G2), fucose (F), bisecting N-acetylglucosamine (N) and N-acetylneuraminic acid (S) residues. Structural schemes: blue square (N-acetylglucosamine), red triangle (fucose), green circle (mannose), yellow circle (galactose), and purple diamond (N-acetylneuraminic acid). *^a^*SwissProt entry number.

| glycan species | Structural schemes | IgG1 P01857^a^ (EEQYNSTYR) | IgG2 P01859^a^ (EEQFNSTFR) | IgG4 P01861^a^ (EEQFNSTYR) |
| --- | --- | --- | --- | --- |
|  |  | [M + 3H]^3+^ | [M +3 H]^3+^ | [M +3 H]^3+^ |
| No glycan |  | 397.1755 | 386.5123 | 391.8439 |
| G0F | 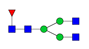 | 878.6868 | 868.0236 | 873.3552 |
| G1F | 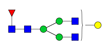 | 932.7044 | 922.0412 | 927.3728 |
| G2F | 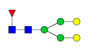 | 986.7220 | 976.0588 | 981.3904 |
| G0FN | 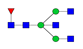 | 946.3800 | 935.7167 | 941.0483 |
| G1FN | 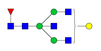 | 1000.3976 | 989.7343 | 995.0659 |
| G2FN | 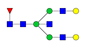 | 1054.4152 | 1043.7519 | 1049.0835 |
| G1FS | 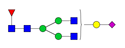 | 1029.7362 | 1019.0730 | 1024.4046 |
| G2FS | 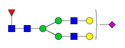 | 1083.7538 | 1073.0906 | 1078.4222 |
| G0 | 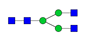 | 830.0009 | 819.3376 | 824.6692 |
| G1 | 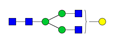 | 884.0185 | 873.3552 | 878.6868 |
| G2 | 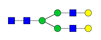 | 938.0361 | 927.3728 | 932.7044 |
| G0N | 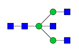 | 897.6940 | 887.0307 | 892.3623 |
| G1N | 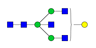 | 951.7116 | 941.0483 | 946.3800 |
| G2N | 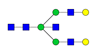 | 1005.7292 | 995.0659 | 1000.3976 |
| G1S | 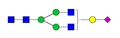 | 981.0503 | 970.3870 | 975.7186 |
| G2S | 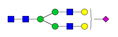 | 1035.0679 | 1024.4046 | 1029.7362 |

**Table S3.** **The variable importance in projection (VIP) score from PLS-DA to distinguish AIP, PDAC, and control.** The PLS-DA conducted based on 16 IgG1 glycoforms, 9 IgG2 glycoforms, and 9 IgG4 glycoforms.

| Ranked by the | Glycoform | VIP Score | | |
| --- | --- | --- | --- | --- |
| relative abundance |  |  |  |  |
| IgG1 |  | AIP vs. Control | PDAC vs. Control | AIP vs. PDAC |
| 1 | G0F | 1.1554 | 1.4399 ☆ | 1.4209 ☆ |
| 2 | G1F | 1.2086 ☆ | 0.4279 | 1.2384 |
| 3 | G2F | 1.1682 | 1.3191 | 1.2538 |
| 4 | G2FS | 0.9775 | 1.2655 | 1.3081 |
| 5 | G1FN | 0.8071 | 0.6308 | 0.0150 |
| 6 | G0FN | 0.7604 | 1.2219 | 1.1179 |
| 7 | G1 | 1.1155 | 1.2019 | 1.2194 |
| 8 | G2 | 0.8363 | 0.9739 | 0.9082 |
| 9 | G0 | 0.7489 | 1.0449 | 1.4001 |
| 10 | G1FS | 0.9310 | 0.9774 | 0.3606 |
| 11 | G1N | 0.8272 | 0.4521 | 1.0043 |
| 12 | G2N | 0.8291 | 0.3273 | 1.1550 |
| 13 | G2S | 0.1553 | 0.8316 | 0.9421 |
| 14 | G2FN | 0.9187 | 0.8165 | 0.4033 |
| 15 | G0N | 1.3956 | 0.6278 | 0.3460 |
| 16 | G1S | 1.4476 | 1.4166 | 0.0000 |
| IgG2 |  |  |  |  |
|  |  | AIP vs. Control | PDAC vs. Control | AIP vs. PDAC |
| 1 | G0F | 1.3716 ☆ | 1.4004 ☆ | 1.2746 ☆ |
| 2 | G1F | 1.3561 | 0.6012 | 0.9969 |
| 3 | G2F | 0.0503 | 1.2526 | 1.0925 |
| 4 | G2FS | 0.9187 | 1.3908 | 1.2141 |
| 5 | G1FS | 1.3903 | 0.3951 | 1.1587 |
| 6 | G0FN | 0.8745 | 1.4131 | 1.2385 |
| 7 | G1FN | 0.1584 | 0.0613 | 0.0880 |
| 8 | G2FN | 0.5742 | 0.8153 | 0.8744 |
| 9 | G0 | 1.1749 | 0.5939 | 0.2554 |
| IgG4 |  |  |  |  |
|  |  | AIP vs. Control | PDAC vs. Control | AIP vs. PDAC |
| 1 | G0F | 1.0398 | 1.2311 ☆ | 1.2538 |
| 2 | G1F | 1.1564 ☆ | 1.1885 | 1.3021 ☆ |
| 3 | G2F | 1.0712 | 1.0554 | 0.3202 |
| 4 | G2FS | 1.1081 | 1.1087 | 1.2648 |
| 5 | G0FN | 0.8341 | 0.9038 | 0.5945 |
| 6 | G1FN | 1.1441 | 1.2364 | 0.7744 |
| 7 | G1FS | 0.9614 | 0.3274 | 1.0283 |
| 8 | G2FN | 0.1786 | 1.0236 | 1.0753 |
| 9 | G0 | 1.1159 | 0.4779 | 0.9294 |
| **☆:** Glycoforms of IgG with the highest VIP score and the relative abundance >5%. | | | | |

**
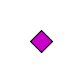

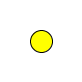

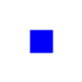

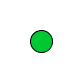
Table S4. Reproducibility of the analytical method for glycoform identification.** The G0 ratio (G0F/G1F) and S ratio (G2FS/G1F) of IgG1, IgG2, and IgG4 were compared, the RSDs were determined from three independent experiments comprising three replicates each.

| Glycoform |  | G0F of IgG1 | G2FS of IgG1 | G1F of IgG1 | G0F of IgG2 | G2FS f IgG2 | G1F f IgG2 | G0F of IgG4 | G2FS of IgG4 | G1F of IgG4 |
| --- | --- | --- | --- | --- | --- | --- | --- | --- | --- | --- |
| Difference between intraday | |  |  |  |  |  |  |  |  |  |
|  | Day 1 |  |  |  |  |  |  |  |  |  |
|  | Ave. | 8289.67 | 15959.67 | 15373.33 | 11738.33 | 10364.67 | 22524.00 | 1141.00 | 1520.67 | 1797.33 |
|  | Std. | 499.78 | 740.84 | 788.07 | 253.08 | 420.76 | 2575.09 | 61.78 | 114.49 | 205.10 |
|  | RSD (%) | 6.03 | 4.64 | 5.13 | 2.16 | 4.06 | 11.43 | 5.41 | 7.53 | 11.41 |
|  | Day 2 |  |  |  |  |  |  |  |  |  |
|  | ave. | 10647.33 | 13273.33 | 12300.33 | 12603.00 | 8254.67 | 20862.67 | 1150.33 | 1099.00 | 1747.33 |
|  | std. | 336.40 | 662.83 | 71.58 | 277.51 | 715.65 | 2308.81 | 43.70 | 77.98 | 70.79 |
|  | RSD. | 3.16 | 4.99 | 0.58 | 2.20 | 8.67 | 11.07 | 3.80 | 7.10 | 4.05 |
|  | Day 3 |  |  |  |  |  |  |  |  |  |
|  | ave. | 10222.33 | 12428.33 | 14596.33 | 13720.33 | 11711.33 | 21273.67 | 1447.67 | 1490.00 | 2218.00 |
|  | std. | 595.11 | 640.61 | 1135.77 | 866.15 | 337.80 | 1054.03 | 41.00 | 38.22 | 150.66 |
|  | RSD (%) | 5.82 | 5.15 | 7.78 | 6.31 | 2.88 | 4.95 | 2.83 | 2.57 | 6.79 |
| Difference between interday | |  |  |  |  |  |  |  |  |  |
|  | Ave. | 9719.78 | 13887.11 | 14090.00 | 12687.22 | 10110.22 | 21553.44 | 1246.33 | 1369.89 | 1920.89 |
|  | Std. | 1136.55 | 1653.16 | 1529.96 | 977.43 | 1513.79 | 2203.79 | 150.83 | 209.12 | 260.41 |
|  | RSD (%) | 11.69 | 11.90 | 10.86 | 7.70 | 14.97 | 10.22 | 12.10 | 15.27 | 13.56 |

Relative standard deviation (RSD)

**Supplementary Figures:**


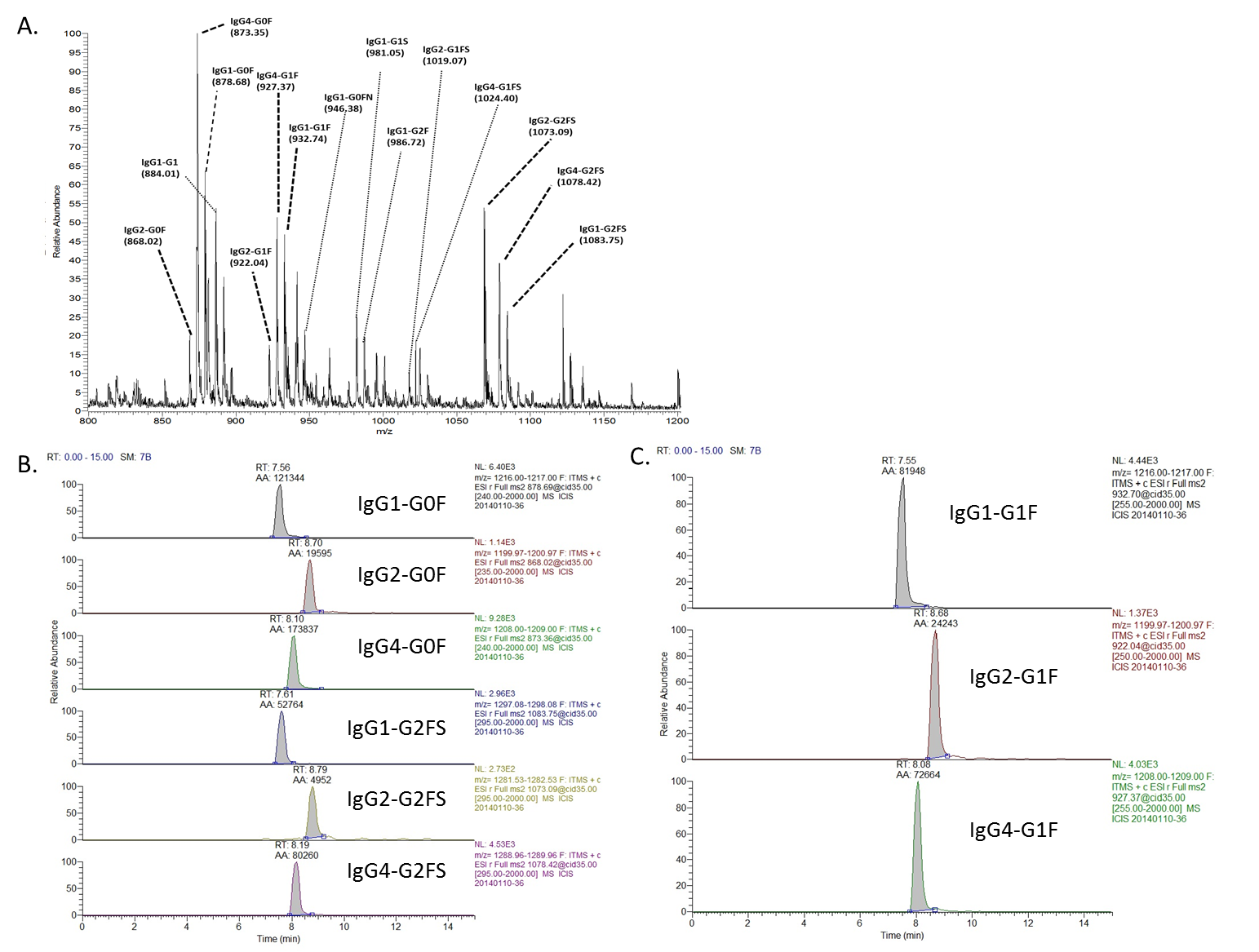


**Figure S1. Glycosylation profiling of IgG and relative quantification of G0F, G1F, and G2FS of IgG1, gG2, and IgG4.** (A) MS spectrum of the tryptic IgG-Fc glycopeptides. (B) The extracted ion chromatograms (XIC) with peak areas for G0F and G2FS of IgG1, IgG2, and IgG4. (C) The extracted ion chromatograms (XIC) with peak areas for G1F of IgG1, IgG2, and IgG4.


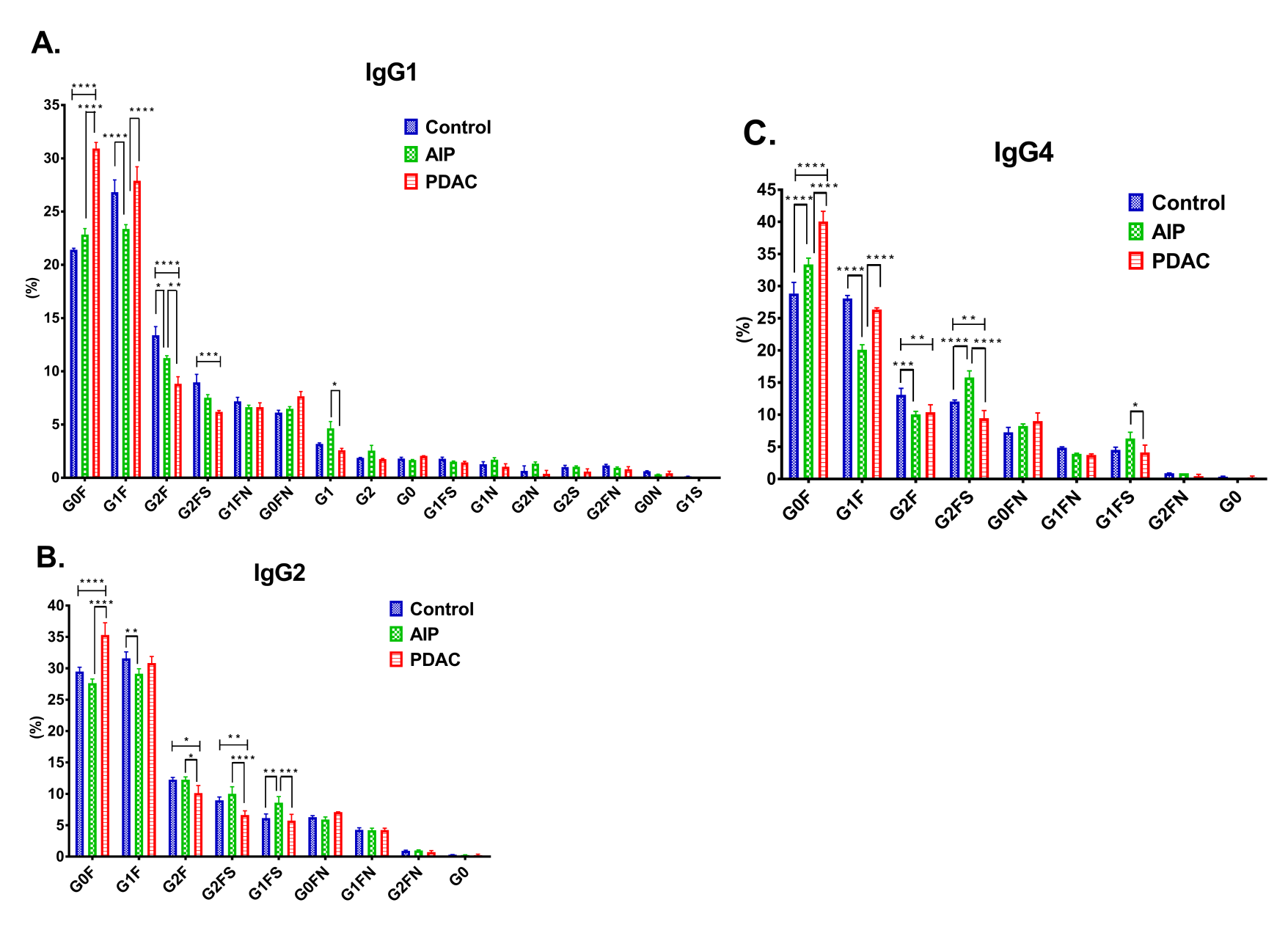


**Figure S2. Glycosylation of IgG1, IgG2 and IgG4 in pooled sera from controls, AIP patients and PDAC patients.** The glycosylation differences between the controls (blue, n=57), patients with AIP (green, n=86), and patients with PDAC (red, n=115) are reported as relative ratios (%). The differences were determined via a two-way ANOVA followed by a Bonferroni test for multiple comparisons. The three replicates are reported as the mean and standard deviation (SD). (A) The glycoforms analyzed according to the glycopeptide profiles are detected from the IgG1 Fc N-glycosylation. (B) The glycoforms are analyzed according to glycopeptide profiles from the IgG2 Fc N-glycosylation. (C) The glycoforms are analyzed according to the glycopeptide profiles from the IgG4 Fc N-glycosylation. p-values: *p<0.05; **p<0.01; ***p<0.001; ****p<0.0001.


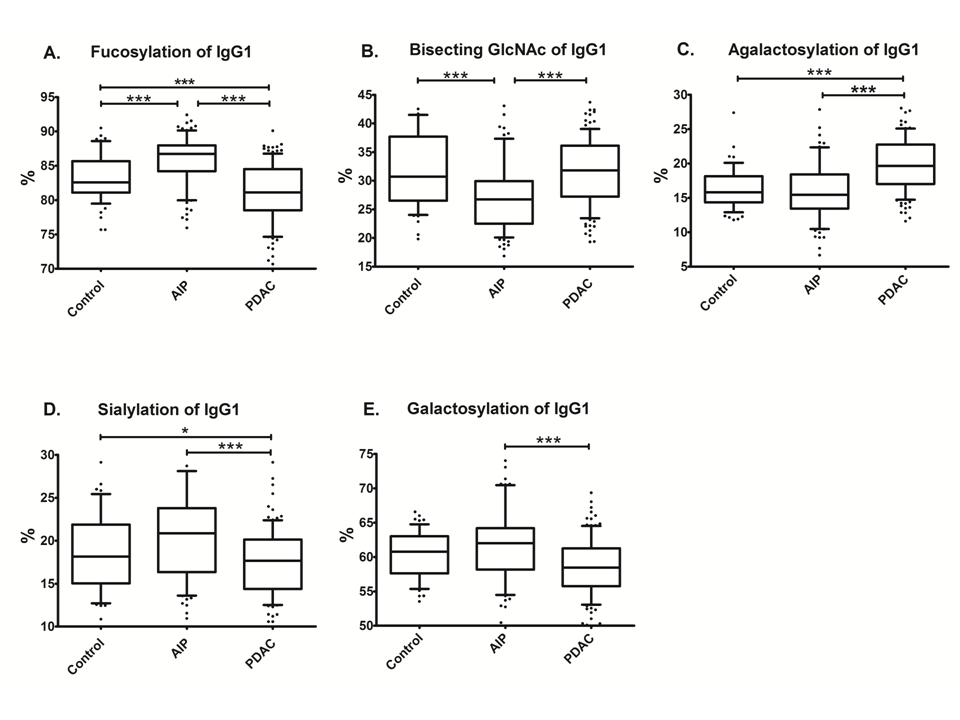


**Figure S3-I. IgG1 Fc N-glycan (A) fucosylation, (B) bisecting GlcNAc, (C) agalactosylation, (D) sialylation, and (E) galactosylation in controls (n=57) and patients with PDAC (n=115) or AIP (n=86).** The differences were determined via a one-way ANOVA followed by a Bonferroni test for multiple comparisons. The boxes extend from the 25th to the 75th percentiles, and the horizontal bars represent the median. The whiskers are drawn down to the 10th percentile and up to the 90^th^ percentile. The points below and above the whiskers are drawn as dots; p-values: *p<0.05; **p<0.01; ***p<0.001.


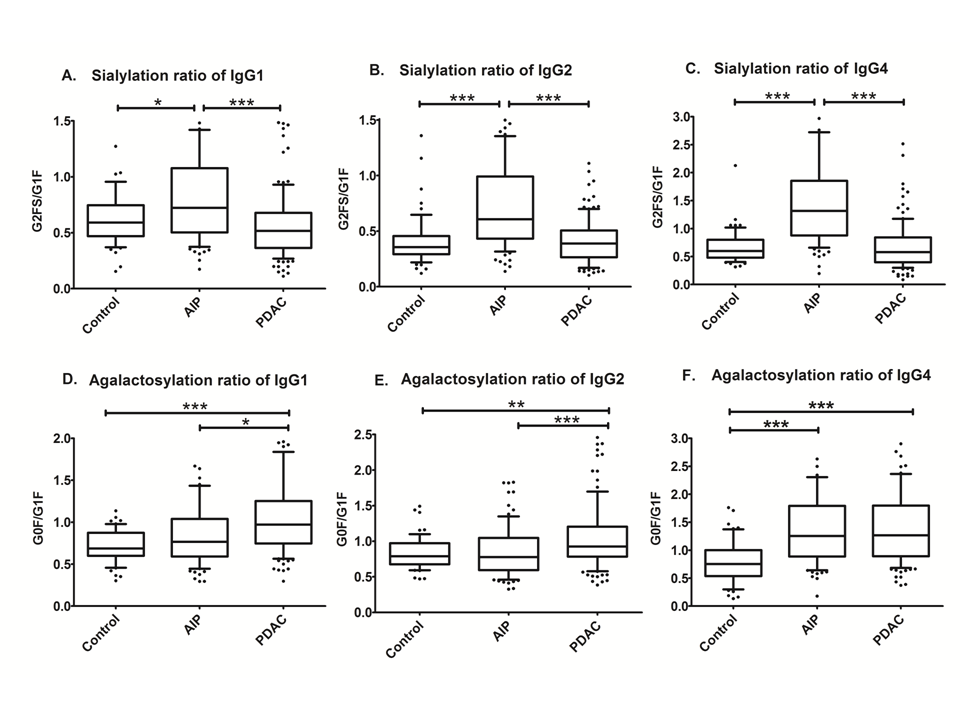


**Figure S3-II. The agalactosylation ratio and sialylation ratio of the three IgG subclasses (IgG1, IgG2 and IgG4) within the three groups of individual sera (controls, PDAC patients and AIP patients).** The three groups of patients include controls (n=57), PDAC patients (n=115), and AIP patients (n=86). The differences were determined via a one-way ANOVA followed by the Bonferroni test for multiple comparisons. The boxes extend from the 25th to 75th percentiles, and the horizontal bars represent the median. The whiskers are drawn down to the 10th percentile and up to the 90^th^ percentile. Points below and above the whiskers are drawn as dots; p-values: *p<0.05; **p<0.01; ***p<0.001; (A) The sialylation ratio of IgG1 among the three groups of patients. (B) The sialylation ratio of IgG2 among the three groups of patients. (C) The sialylation ratio of IgG4 among the three groups of patients. (D) The agalactosylation ratio of IgG1 among the three groups of patients. (E) The agalactosylation ratio of IgG2 among the three groups of patients. (F) The agalactosylation ratio of IgG4 among the three groups of patients.


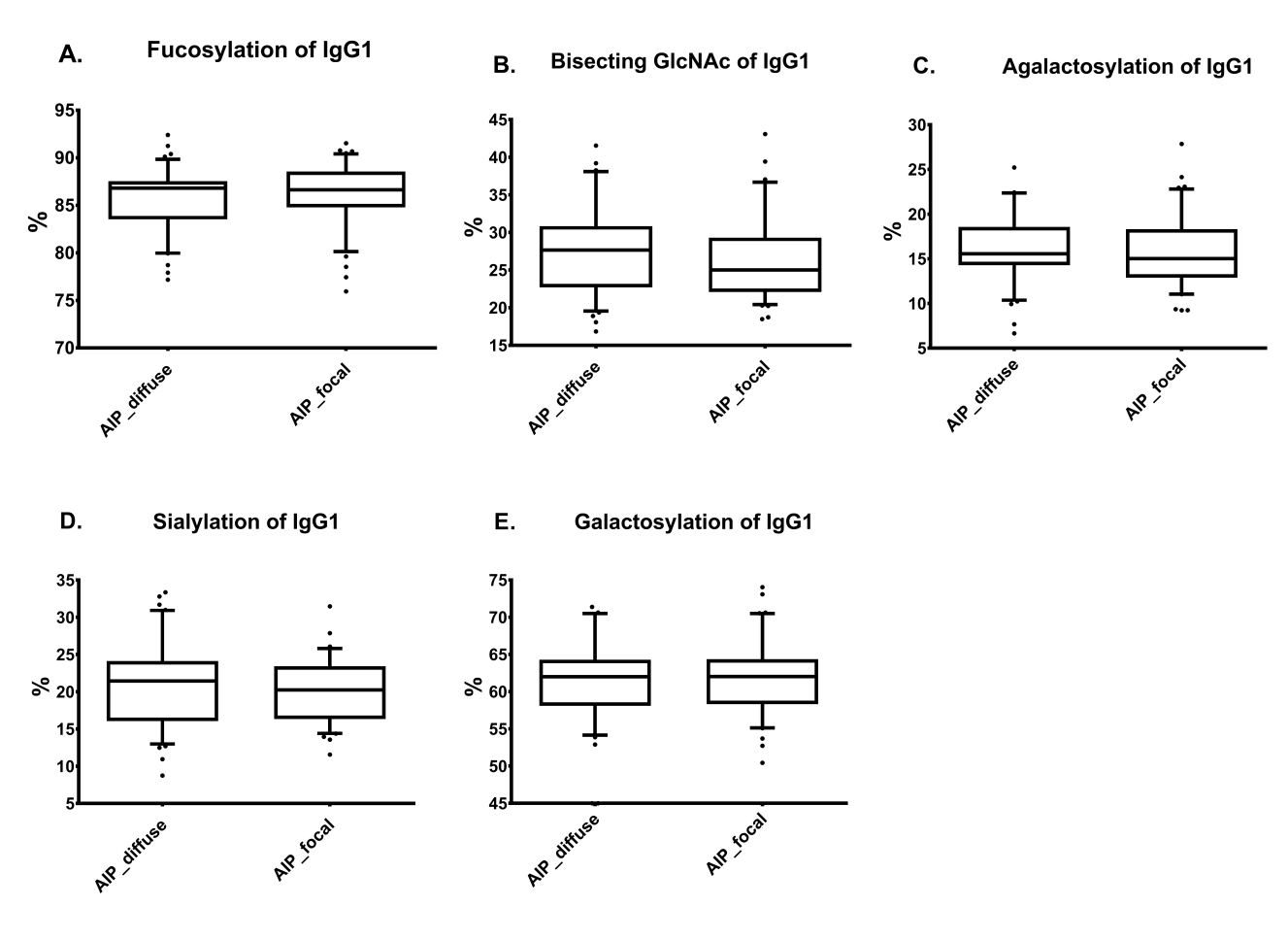


**Figure S4-I. IgG1 Fc N-glycan (A) fucosylation, (B) bisecting GlcNAc, (C) agalactosylation, (D) sialylation, and (E) galactosylation in diffuse type AIP patients (n=45) and focal type AIP patients (n=41).** The differences were determined via t-test. All statistical tests were two-tailed, with statistical significance set at P <0.05. The boxes extend from the 25th to the 75th percentiles, and the horizontal bars represent the median. The whiskers are drawn down to the 10th percentile and up to the 90th percentile. The points below and above the whiskers are drawn as dots.


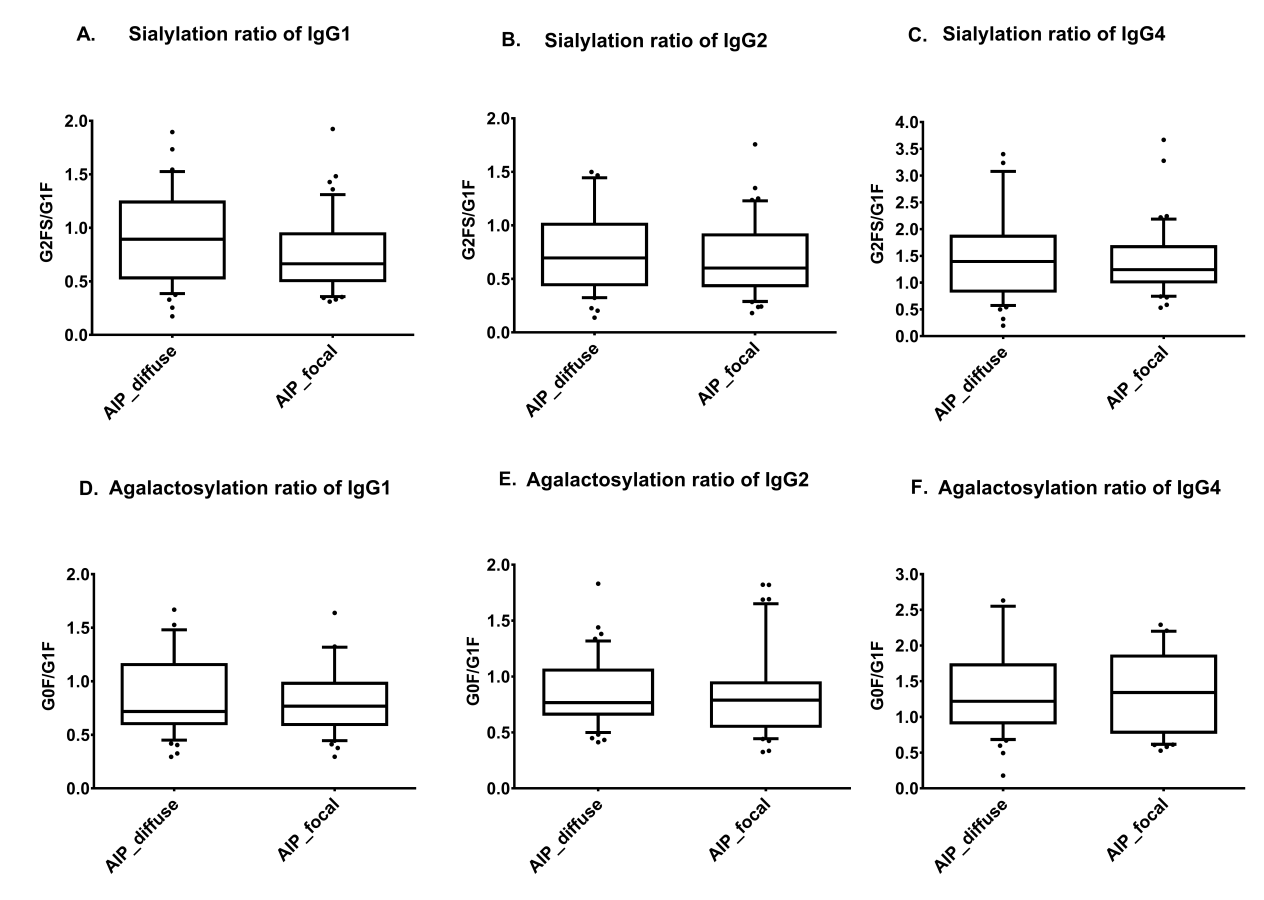


**Figure S4-II. The agalactosylation ratio and sialylation ratio of the three IgG subclasses (IgG1, IgG2 and IgG4) in the diffuse type AIP patients (n=45) and focal type AIP patients (n=41).** (A) The sialylation ratio of IgG1. (B) The sialylation ratio of IgG2. (C) The sialylation ratio of IgG4. (D) The agalactosylation ratio of IgG1. (E) The agalactosylation ratio of IgG2. (F) The agalactosylation ratio of IgG4. The differences were determined via t-test. All statistical tests were two-tailed, with statistical significance set at P <0.05. The boxes extend from the 25th to the 75th percentiles, and the horizontal bars represent the median. The whiskers are drawn down to the 10th percentile and up to the 90th percentile. The points below and above the whiskers are drawn as dots.


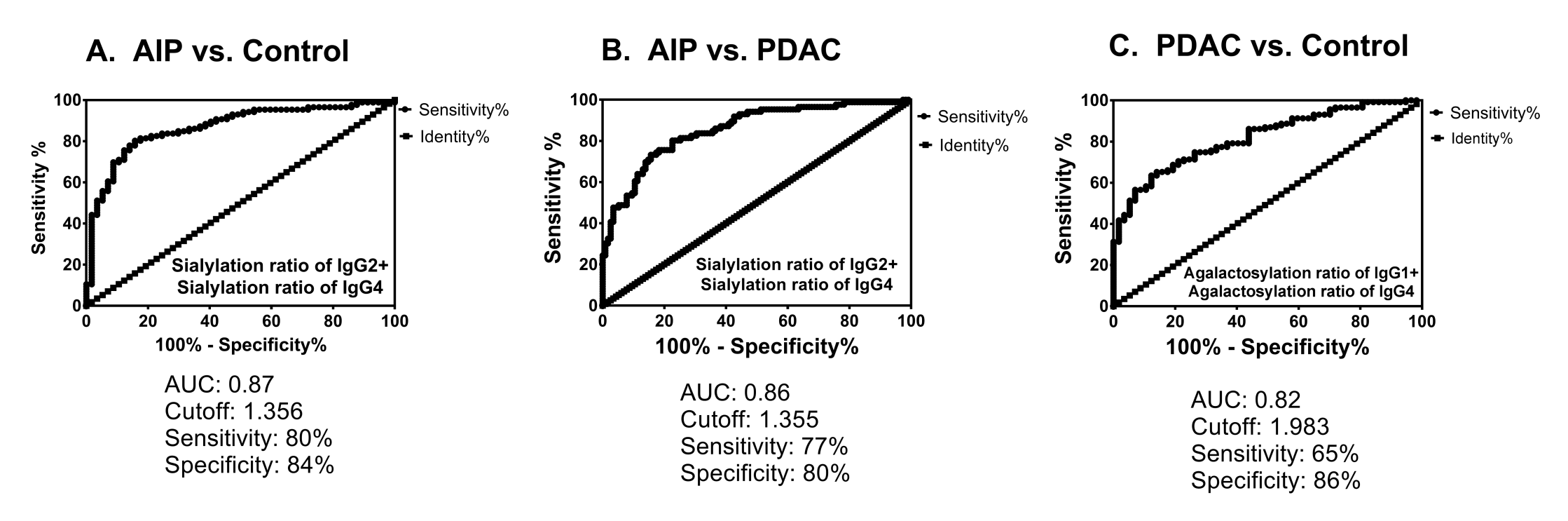


**Figure S5. ROC curve for discrimination among the control, PDAC and AIP groups.** The AUC, cutoff, sensitivity and specificity are shown for each ROC curve. (A) The ROC curve for the sum of the sialylation ratios of IgG2 and IgG4 to discriminate AIP from controls. (B) The ROC curve of the sum of the sialylation ratios of IgG2 and IgG4 to discriminate AIP from PDAC. (C) The ROC curve of the sum of the agalactosylation ratios of IgG1 and IgG4 to discriminate PDAC from controls.

**
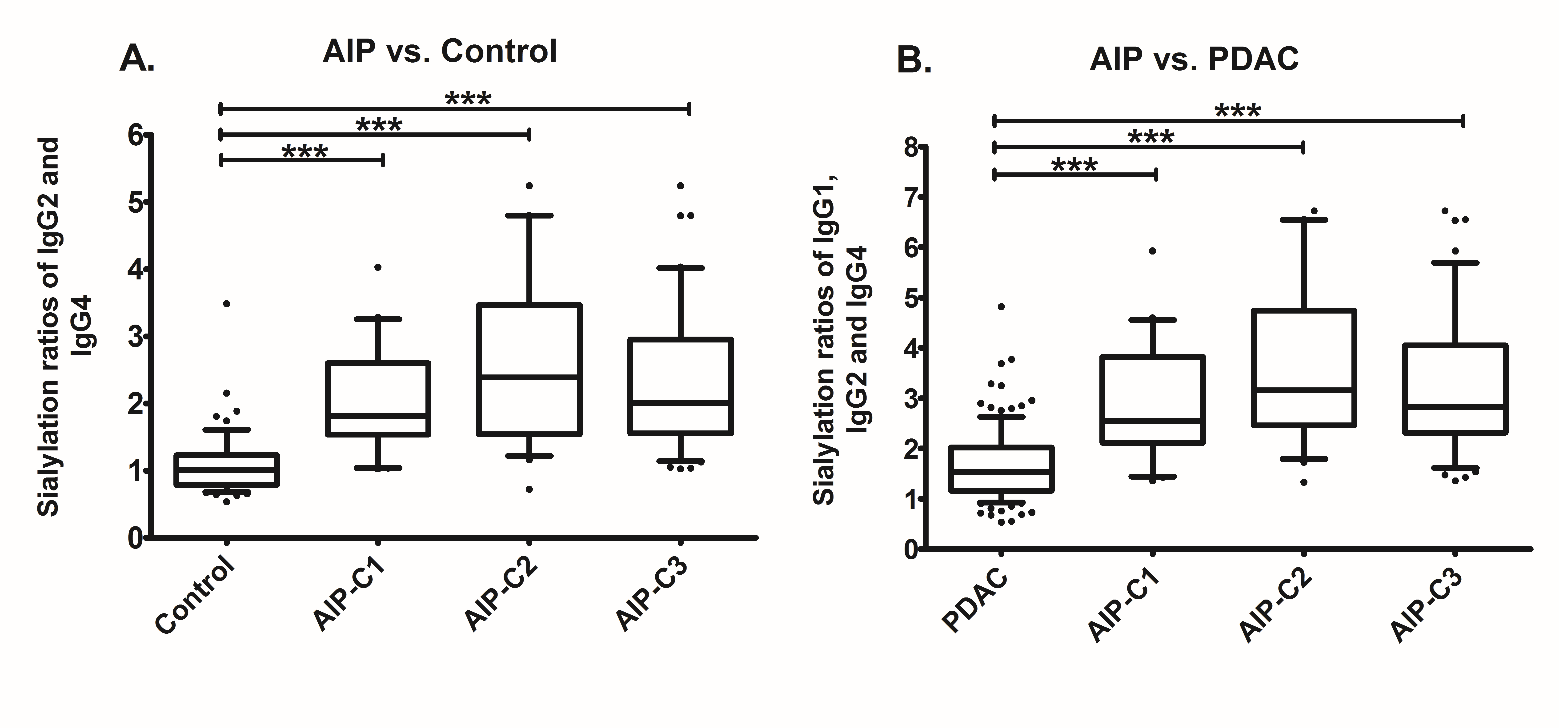
**

**Figure S6. The sialylation ratios in AIP patients with different serum IgG4 concentrations.** AIP-C1 (140 mg/dl<Serum IgG4<280 mg/dl, n=22), AIP-C2 (serum IgG4>280mg/dl, n=22), AIP-C3 (IgG4/IgG>10% , n=43). The differences were determined via a one-way ANOVA followed by the Bonferroni test for multiple comparisons. The boxes extend from the 25th to 75th percentiles, and the horizontal bars represent the median. The whiskers are drawn down to the 10th percentile and up to the 90th percentile. Points below and above the whiskers are drawn as dots; ***p<0.001; (A) The sialylation ratio of IgG2 and IgG4 among the AIP-C1~C3 and control. (B) The sialylation ratio of IgG1, IgG2, and IgG4 among the AIP-C1~C3 and PDAC.
